# Protein Kinase G Induces an Immune Response in Cows Exposed to* Mycobacterium avium* Subsp.* paratuberculosis*

**DOI:** 10.1155/2018/1450828

**Published:** 2018-01-18

**Authors:** Horacio Bach, Melissa Richard-Greenblatt, Eviatar Bach, Marcelo Chaffer, Wanika Lai, Greg Keefe, Douglas J. Begg

**Affiliations:** ^1^Department of Medicine, Division of Infectious Diseases, University of British Columbia, 410-2660 Oak St., Vancouver, BC, Canada V6H 3Z6; ^2^Department of Health Management, Atlantic Veterinary College, University of Prince Edward Island, 550 University Avenue, Charlottetown, PE, Canada C1A 4P3; ^3^Sydney School of Veterinary Science, School of Life and Environmental Sciences, The University of Sydney, 425 Werombi Rd, Camden, NSW 2570, Australia

## Abstract

To establish infection, pathogens secrete virulence factors, such as protein kinases and phosphatases, to modulate the signal transduction pathways used by host cells to initiate immune response. The protein MAP3893c is annotated in the genome sequence of* Mycobacterium avium* subspecies* paratuberculosis *(MAP), the causative agent of Johne's disease, as the serine/threonine protein kinase G (PknG). In this work, we report that PknG is a functional kinase that is secreted within macrophages at early stages of infection. The antigen is able to induce an immune response from cattle exposed to MAP in the form of interferon gamma production after stimulation of whole blood with PknG. These findings suggest that PknG may contribute to the pathogenesis of MAP by phosphorylating macrophage signalling and/or adaptor molecules as observed with other pathogenic mycobacterial species.

## 1. Introduction

Each year the dairy industry suffers significant economic losses from the infectious agent* Mycobacterium avium *subspecies* paratuberculosis *(MAP) [1]. The bacterium causes cattle and other ruminants a fatal gastrointestinal disease known as Johne's disease (JD). The National Animal Health Monitoring System estimates the prevalence of JD in US dairy herds at 68% [2]. Animals can remain asymptomatic for many years and shed large numbers of the bacteria in their faeces resulting in a transmission via faecal–oral route to many other cattle before the appearance of any clinical presentation in the herd [3].

In addition, the presence of MAP in dairy products [4, 5] represents a potential concern to human health. Recent advances in diagnostics have enabled the isolation of MAP from intestinal tissues [6], blood [7, 8], and breast milk [9] of Crohn's disease (CD) patients. We have also observed higher antibody titers against PknG in CD patient sera, suggesting an early exposure or the presence of MAP in these patients [10]. Collectively, the association of MAP with CD patients, the reported clinical and pathological similarities in the gut of CD and JD, and their association with MAP [11] have led to the hypothesis that this bacteria may be an etiological agent for CD [12].

Currently, there is limited knowledge on the underlying mechanisms involved in MAP pathogenesis. MAP, as other pathogenic mycobacteria, is able to effectively block the immunological response of host macrophages, contributing to the bacterium's ability to successfully survive and replicate inside these cells [13, 14]. The best characterized of the mycobacterial species is* M. tuberculosis *(Mtb), the bacterium responsible for tuberculosis. The genome of Mtb encodes for 11 serine/threonine protein kinases, nine of which are localized in the plasma membrane [15]. Of the remaining soluble proteins, protein kinase G (PknG) is important for the survival of Mtb* in vivo* [16]. With the vaccine strain* Mycobacterium bovis *Bacille Calmette-Guérin (BCG), Walburger and colleagues observed PknG to be secreted within macrophage phagosomes and effectively inhibit phagosome–lysosome fusion leading to enhanced survival of the bacterium [17]. To date, this observation is limited to* M. bovis *BCG, although this kinase is suggested to play a similar role in the virulence of* M. marinum* [18]. In addition to its proposed contribution to pathogenicity, PknG has also been shown to have an intracellular catalytic activity, such as the regulation of the glutamate metabolism and the TCA cycle in Mtb [17, 19] and* Corynebacterium glutamicum* [20]. It is also plausible that the observed dependence of intracellular survival of pathogenic mycobacteria on PknG is due to its regulation of metabolic processes that may indirectly influence virulence. Recently, it has been shown that PknG mediates the adaptation of* M. smegmatis* and Mtb to acidic environments [21].

As MAP PknG shares close homology with Mtb and* M. bovis *BCG PknG it was of interest to biochemically characterize the enzyme and further verify its role in MAP pathogenesis. Previous work also identified PknG as contributing to the survival of the bacterium in bovine macrophages and calves; however, polar effects in this strain were not ruled out [22]. In the current study we demonstrate that MAP3893c, which is annotated in the MAP genome for PknG, encodes for a functional kinase that is secreted upon infection of human macrophage cells. Furthermore, PknG is able to induce an immune response in cattle exposed to MAP during early infection.

## 2. Materials and Methods

### 2.1. Strains, Cell Lines, Plasmid Construction, and Growth Conditions

*pknG* amplified from genomic MAP strain k-10 (ATCC BAA-968) was cloned into pET-30b using* Eco*RI and* Xho*I restriction sites using the forward primer 5′-ATAGAATTCAAAATGGCCGAGCCGGACAA, whereas 5′-ATACTCGAGTCAGAACGTGCTGGTGGG was used as a reverse primer. The plasmid was transformed into* E. coli* strain DH5*α*, and, once confirmed through sequencing, it was transformed into* E. coli* BL21(DE3) for protein production. Both* E. coli* strains were grown in Luria-Bertani (LB) broth supplemented with kanamycin (50*μ*g/mL, Sigma). MAP was cultured in 7H9 medium (BD) supplemented with 0.05% Tween-80 (Sigma), 2 mg/L of Mycobactin J (Allied Monitor), and 10% oleic acid-albumin-dextrose-catalase (OADC, BD) and was grown in rolling bottles at 37°C.

The human-derived monocytic cell line, THP-1 (ATCC TIB-202), was chosen as a model for infection, which has been previously used by others to evaluate MAP infections [23, 24]. THP-1 cells were grown in RPMI 1640 (Sigma) supplemented with 1% L-glutamine (Stem Cell Technologies, Vancouver, British Columbia, Canada), 100*μ*g of streptomycin/mL and 100 U of penicillin/mL (Stem Cell Technologies), 0.1% amphotericin B (Fungizone, Invitrogen), and 10% foetal calf serum (FCS, Sigma). FCS (10%) and 1% L-glutamine supplemented RPMI medium was used for infecting THP-1 cells with MAP without the addition of penicillin, streptomycin, or amphotericin B.

The production of recombinant PknG is detailed in Supplementary Information (available here).

### 2.2. *In Vitro* Kinase Assay

The* in vitro* kinase reactions contained 20 mM Tris-HCl pH 7.4, 5 mM MgCl_2_, 5 mM MnCl_2_, 1 mM DTT, 2 mM sodium orthovanadate, 1*μ*g PknG recombinant protein, and 1*μ*g myelin basic protein (MBP, Sigma). Each reaction was initiated by the addition of 10*μ*Ci of*γ*-[^32^P] (PerkinElmer) and was left at room temperature for 30 min. Following the incubation period, the reactions were stopped with sodium dodecyl sulphate- (SDS-) loading sample buffer and were boiled for 7 min at 95°C. The samples were resolved using a SDS-12% polyacrylamide gel electrophoresis (SDS-12% PAGE). ^32^P-labelled proteins were detected using a Phosphorimager SI (Molecular Dynamics).

### 2.3. THP-1 Cells Infection

Prior to infection, MAP cells were washed with 7H9 medium and labelled with 10*μ*g of Rhodamine 6G (stock solution 1 mg/mL in DMSO, Sigma) dissolved in 1 mL of fresh 7H9 medium and rocked for 1 h at room temperature. For the negative control, 500*μ*L of a stationary culture of MAP (OD_600_ = 0.98) was killed by adding 100*μ*g/mL of kanamycin and was left rocking overnight at 37°C. Labelled live and killed cells were then washed repeatedly (×3) with Hank's buffer (Sigma) and opsonized with 10% human AB^+^ serum (Gibco) for 30 min at 37°C [25]. Details about the cell infection are explained in Supplementary Information.

### 2.4. Immunostaining and Fluorescence Microscopy

THP-1 macrophages infected with MAP as mentioned earlier were processed as published [26]. The details of the protocol used in this experiment are in the Supplementary Information.

### 2.5. THP-1 Cell Lysate Immunoprecipitation

Preliminary experiments showed that the anti-Mtb PknG primary antibody detected MAP PknG using recombinant PknG as the control (data not shown). Immunoprecipitation was performed similarly to methods previous described [25] and explained in Supplementary Information.

### 2.6. Immunological Responses from Cattle

The animal ethics committee of the University of Prince Edward Island (PEI), Canada, approved all of the animal procedures used in this study. The cattle were sourced from three dairy farms on PEI. The farms were selected based on their JD history. One farm was known to have a moderate to high prevalence of JD, another farm was with no recorded history of clinical cases of JD, but a low prevalence of MAP infection, and the third farm was identified with greater than 8 years of negative antibody ELISA testing and no recorded cases of JD or MAP infection. Twenty animals were sampled from the high prevalence farm and 10 animals each from the low prevalence and JD-free farm. At sampling, whole blood and serum samples were collected along with a faecal sample from each animal. The serum sample was tested for MAP-specific antibodies using a commercially available kit (Institut Porquier, IDEXX) following the manufacturer's instructions. The data are presented as S/P%, which was calculated as

(1) $$\begin{array}{c} \frac{\left(\text{sample}-\text{OD negative control}\right)}{\text{(OD positive control}-\text{OD negative control)}}\times 100. \end{array}$$

Faecal samples were processed as described by VetAlert Johne's Real-Time PCR kit (Tetracore), targeting the* hspX* gene as described previously [27].

Interferon gamma (IFN-*γ*) production specific to PknG was done on the whole blood sample. The blood from each animal was stimulated with 10*μ*g/mL recombinant PknG antigen, which was endotoxin purified (High Capacity Endotoxin Removal Column, Pierce), purified protein derivative of MAP (PPDJ, USDA-National Veterinary Services Laboratories, Ames, Iowa) at 30*μ*g/mL (antigen control), Pokeweed mitogen (PWM, Sigma) at 5*μ*g/mL (positive control), and PBS (negative control). 225*μ*L of blood was added to 25*μ*L of each of the antigen, mitogen, or PBS. The stimulations were incubated in a humidified chamber at 35°C for approximately 24 h. After incubation, 100*μ*L of the plasma was removed from each stimulation and frozen at −20°C until required. Assessment of IFN-*γ* concentration per sample was completed using a commercial sandwich ELISA (ID Screen Ruminant IFN-*γ* kit, IDVET, Montpellier, France) as previously described [27]. The data are presented as S/P% as described above.

### 2.7. Measurement of Specific PknG Antibodies

A standard ELISA was used to measure the levels of antibodies against PknG in animal sera using recombinant protein and following published protocols [10].

### 2.8. Statistical Analysis

Results were analyzed using a* t*-test analysis. Data was processed using GraphPad Prism version 6.00 (CA, USA). Results were considered significant when the *p* value was <0.05.

## 3. Results

### 3.1. *In Silico* Analysis of PknG among Mycobacteria

**Figure 1:**
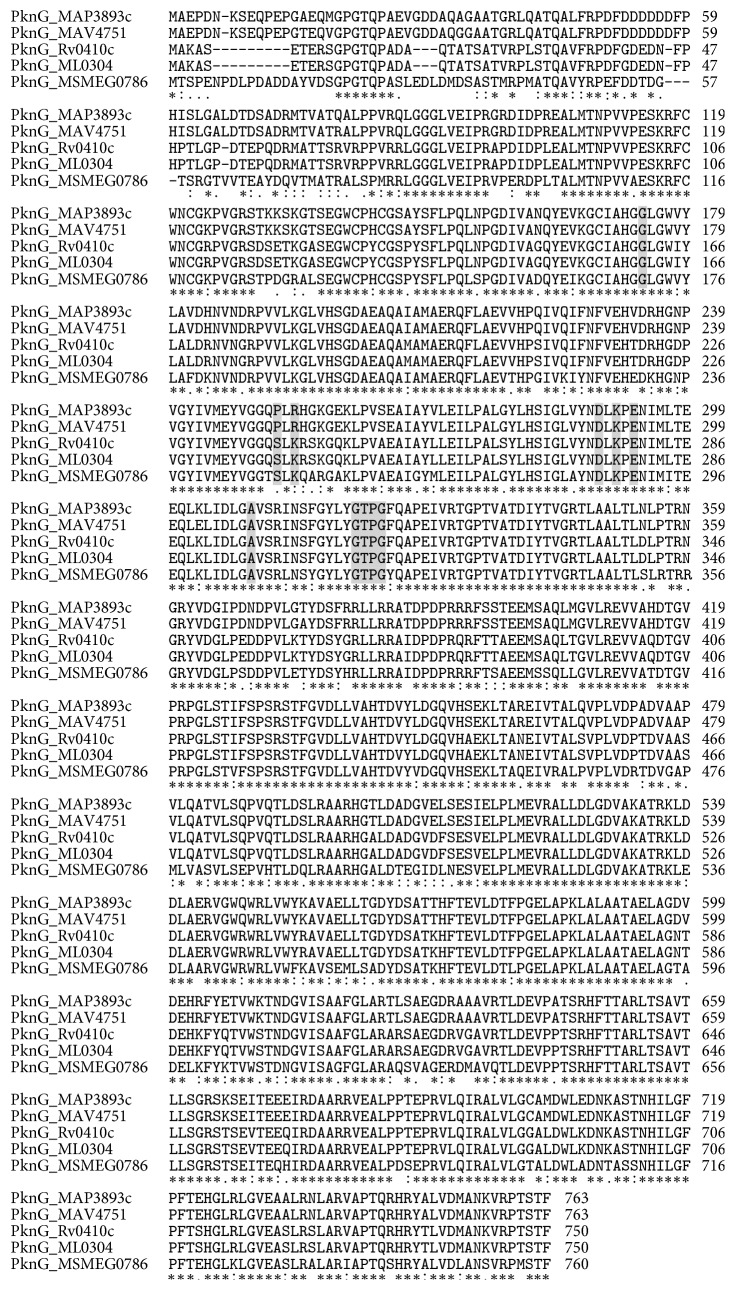
MAP PknG alignment to mycobacterial PknGs. Selected mycobacterial PknG were aligned to MAP PknG. The PknG from the following* Mycobacterium* strains were aligned (locus tag, identity, similarity):* M. avium *subsp.* paratuberculosis *(MAP3893c),* M. avium *104 (MAV4751, 86%. 92%),* M. tuberculosis *(Rv0410c, 86%, 91%),* M. leprae* (ML0304, 81%, 88%), and* M. smegmatis *(MSMEG0786, 79%, 81%). Letters in gray represent the substrate-binding residues.

Kinases are ubiquitously found in eukaryotic and prokaryotic cells and play an essential role in signal transduction. These signalling proteins can be identified based on a conserved protein kinase domain. Among mycobacteria, a family of “eukaryotic-like” serine/threonine protein kinases exist [15], one of which is PknG. Using the NCBI Basic Local Alignment Search Tool, we identified PknG to be conserved among mycobacteria with homologies indicated in Figure 1.

### 3.2. *In Vitro* PknG Expression and Enzymatic Activity

**Figure 2:**
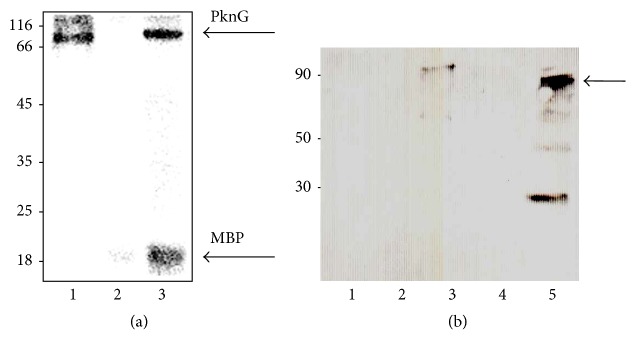
Phosphorylation activity and immunoprecipitation of MAP PknG. (a)* In vitro *phosphorylation of MBP by MAP PknG. Recombinant his-tagged PknG was purified and incubated with [*γ*- ^32^P] ATP and MBP. Samples were separated by SDS-12% PAGE and stained with Coomassie blue followed by visualization by autoradiography. Lanes: 1, recombinant PknG; 2, MBP; 3, recombinant PknG + MBP. Molecular mass markers are indicated on the left in kDa. (b) The immunoprecipitants of infected and noninfected THP-1 cells were resolved by SDS-10% PAGE, electroblotted onto a nitrocellulose membrane, and exposed to rabbit anti-PknG antibodies. Lanes: 1, THP-1 cell-free lysate; 2, BSA (negative control); 3, recombinant PknG; 4, THP-1 cell lysate; 5, MAP-infected THP-1 lysate. Molecular mass markers in kDa; arrow points to the whole PknG.

As the kinase activity of PknG was shown to be essential in blocking phagosome–lysosome fusion [17, 28], we were first interested in examining if MAP* pknG *encoded for a functional kinase. Consequently, recombinant MAP PknG was produced in* E. coli* BL21 and purified using Ni-NTA affinity chromatography. To determine if PknG is active, we conducted an* in vitro* kinase assay to assess PknG's ability to phosphorylate the dephosphorylated myelin basic protein (MBP) used as a universal peptide substrate. As shown in Figure 2, in the presence of PknG, MBP was phosphorylated whereas MBP alone did not undergo autophosphorylation. These findings indicate that MAP PknG possesses* in vitro* kinase activity.

### 3.3. PknG Is Expressed and Secreted* Ex Vivo*

Recently, PknG was discovered as a substrate of the SecA2 secretion system in* Mycobacterium marinum *and the resulting phenotypical defects of a* secA2* transposon mutant were observed to be similar to that described for the Δ*pknG *in other pathogenic mycobacteria [18]. Furthermore, the authors showed that the overexpression of PknG partially restored inhibition of phagosomal maturation in the* secA2 mutant *suggesting not only that PknG is able to be transported by the canonical Sec pathway, albeit with reduced efficiency, but also that PknG-mediated phagosomal maturation arrest is indeed dependent on its secretion. As a result, we further explored whether MAP secretes PknG* ex vivo*. First, we were interested in examining if PknG was indeed expressed during infection of THP-1 cells. As observed in Figure 2(b), we identified a protein corresponding to the molecular weight of PknG in both the lysate of the infected THP-1 cells and the positive control (MAP PknG recombinant protein). In contrast, the lysate obtained from noninfected THP-1 cells did not show any band corresponding to PknG, suggesting the protein is expressed during infection. The additional band of ~30 kDa observed in lane 5 appeared to be a self-cleavage of the protein with unknown activity as reported in a previous study [16].

**Figure 3:**
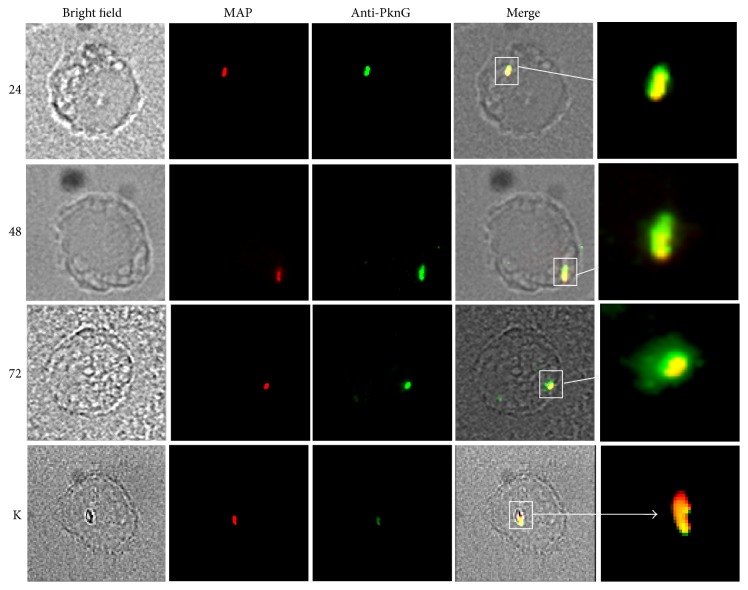
PknG is secreted by MAP during infection. Fluorescence microscopy of infected THP-1 cells. THP-1 cells were infected with rhodamine 6G-labelled bacteria. Samples were obtained at 24, 48, and 72 h after infection and permeabilized with primary and secondary antibodies as described. PknG secretion was observed using fluorescence microscopy, where yellow indicates colocalization of the bacterium (in red) and the Alexa 488-anti-PknG antibodies (in green). Numbers on the left indicate hours. K, killed bacteria. White squares indicate phagosomes inside macrophages.

Although our findings suggest that PknG is expressed by MAP in macrophages, it was of further interest to determine if the kinase is secreted by MAP and may be interfering with host signalling pathways and/or adaptor molecules. As a result we monitored colocalization of PknG at various time points during infection using epifluorescence microscopy. Immunostaining showed that both antibiotic-killed and live MAP cells were readily phagocytosed by the macrophages, as seen by the red fluorescently labelled bacteria (Figure 3). The secretion of PknG within macrophages is visualized by a green halo surrounding the yellow bacterium (red and green) in the overlapping image. The green fluorescence associated with PknG was only observed within macrophages hosting live bacteria, indicating that the presence of cytosolic PknG is dependent on active secretion by MAP rather than the result of bacterial lysis. Furthermore, visual comparison of the various time points of infection suggest that PknG secretion increased following the 24 h time point as the green halo surrounding the bacterium is greater in 48 h and 72 h images. Taken together, the data suggests that PknG expression is elevated at 48 h and remains stable at 72 h.

### 3.4. Immunological Responses from Cattle

**Figure 4:**
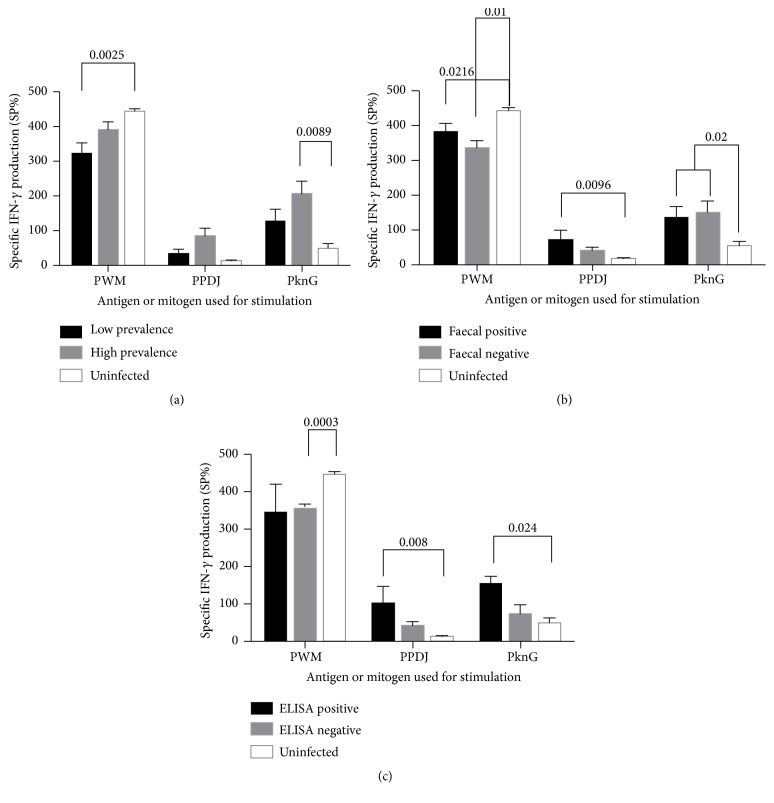
Measurement of IFN-*γ* secreted in whole blood exposed to mycobacterial antigens. Blood was stimulated with Pokeweed mitogen (PWM), PPDJ, or PknG. Cattle were categorized based on (a) prevalence of MAP infection on farms, (b) PCR diagnosis of faecal shedding of MAP, and (c) serotype according to IDEXX ELISA. Bars are representative of the mean ± SE.

If PknG is secreted within macrophage cytoplasm upon MAP infection, it is expected that cattle may have PknG-specific immunity as a result of the exogenous antigen presentation by the Major Histocompatibility Complex (MHC) class II. This specific immunity can be measured by detection of specific antibodies or cytokines in blood after antigen stimulation [29, 30]. Thus, to assess whether lymphocytes have a memory response to PknG, we compared IFN-*γ* levels in cattle from high and low prevalence farms. As hypothesized, the production of IFN-*γ* in whole blood obtained from cattle stimulated with PknG had significantly elevated levels in herds from high prevalence farms compared to low prevalence and uninfected farms (Figure 4(a)). Interestingly, whole blood stimulated with the PPDJ antigen showed comparable levels of IFN-*γ* across all herds. Analysis of cattle further classified by PCR detection of MAP in faeces identified 11 positive (11/30, 37%) and 19 negative animals (19/30, 63%) from the high and low prevalence farms combined. Both PknG and PPDJ stimulated significantly greater IFN-*γ* production from the whole blood of MAP-shedding cattle than that of uninfected herds (Figure 4(b)). However, PCR negative cattle also exhibited levels of IFN-*γ* similar to PCR positive cattle suggesting that a number of PCR negative cattle have subclinical infection (Stage I or II of JD). To further support these findings, we observed that PknG antigen stimulated significantly more IFN-*γ* production in those deemed seropositive in comparison to those deemed seronegative (Figure 4(c)).

**Figure 5:**
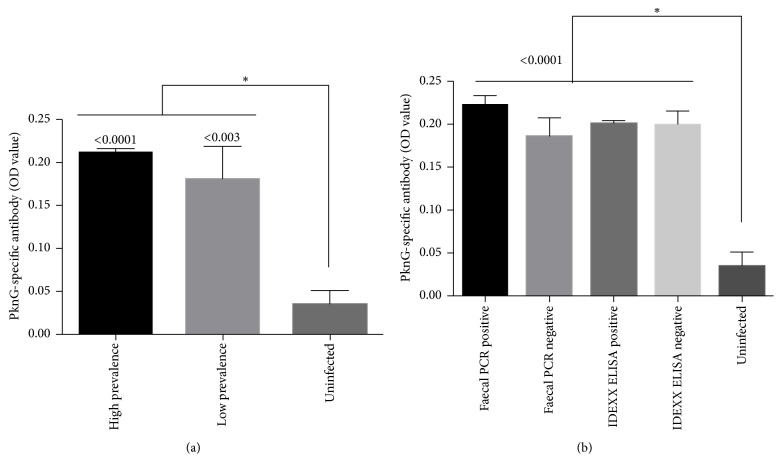
Immunogenicity of PknG in cattle sera. (a) Sera from high and low prevalence or uninfected farms and (b) sera from cattle classified as positive or negative for faecal PCR and IDEXX ELISA. Shown is the mean ± SEM. ^*∗*^*p* value < 0.05.

We further investigated PknG-specific antibody responses from sera collected of these same cattle and observed a significant difference between the MAP-exposed and the uninfected herds, where the MAP-exposed herds had more specific antibody (Figure 5(a)). Similar to our whole blood analysis, differentiating the animals from the exposed herd into faecal PCR and commercial ELISA (IDEXX) positive or negative cohorts indicated that there was no difference in PknG-specific antibody responses (Figure 5(b)). Again, these results may suggest that animals are in the initial stages of infection and may only be shedding MAP intermittently or generating inadequate levels of antibody in the blood.

## 4. Discussion

Mycobacteria, along with a number of other intracellular pathogens, can evade the antimicrobial responses of the host through the secretion of bacterial proteins, such as kinases and phosphatases [26, 31], that can modulate host intracellular signalling pathways. PknG in* M. bovis* is one such example and has been shown to prevent phagosome–lysosome fusion similar to its pathogenic mycobacterial counterparts [17]. However, little is known regarding the role of PknG in the pathogenicity of MAP. In the current study we demonstrated that the open reading frame MAP3893c, a homolog of PknG in mycobacteria, encodes for an active kinase that is secreted during infection.

The distribution of PknG in Actinomycetales has been already reported [32]. A number of studies have also shown that PknG plays a metabolic role in this order. Accumulation of the amino acids glutamine and glutamate was observed in a Mtb* pknG* knockout strain [16], and in later studies in* C. glutamicum *PknG was found to control the activity of the enzyme 2-oxoglutarate dehydrogenase by phosphorylating its substrate OdhI, a protein involved in glutamate synthesis [20]. In addition to its metabolic role in bacteria, PknG was also found to function as a virulence factor as observed by its secretion into the macrophage's cytosol and ability to block phagosome–lysosome fusion [17]. As a result of these findings in* M. bovis, *we hypothesized that PknG may play a similar role in the pathogenicity of MAP. In this work, we show that MAP PknG, similar to* M. bovis*-PknG, is secreted during infection as the protein was detected using immunoprecipitation in the lysate extracts of THP-1 cells infected with MAP.

Analysis of PknG secretion in live bacterium-infected macrophages showed secretion at 24 h followed by a concomitant increase in fluorescence intensity during the full course of the experiment (72 h). Other groups have previously shown that PknG expression in pathogenic mycobacteria is induced by the intracellular environment of the macrophage [17] and further increases at 48 h and 110 h after infection [18]. Despite the essential role for PknG in the survival of* M. bovis*, little remains known as to its secretion mechanism and host substrates. Recently the* M. marinum* SecA2 secretion system was found to be responsible for the secretion of PknG into the intraphagosomal environment and the SecA2 mutant displayed similar defects to that described for the* pknG *knockout strain [18]. To date, no other secretion systems have been described and it remains plausible that MAP uses a similar mechanism.

Walburger et al. observed that the kinase activity of PknG is essential for its ability to prevent phagosome–lysosome fusion [17]. Thus to ensure that PknG was enzymatically active, we analyzed the protein's phosphorylation activity using an* in vitro* kinase assay. The results for the experiment showed that MAP3983c encodes for a functional kinase, as it was able to phosphorylate the universal kinase substrate, MBP. Although no other studies have shown MAP3983c to exhibit kinase activity, these findings were expected as the protein shares high homology with PknG in* M. bovis* and Mtb. Thus, our results suggest that PknG has the potential to interfere with host signalling pathways in a similar fashion to* M. bovis*.

The whole blood responses to the PknG antigen were different to those observed with PPDJ, as animals with no faecal shedding or those seronegative for MAP responded similarly to PknG as positive animals. These findings indicate that the observed immunological response to PPDJ is the result of the cattle's exposure to MAP rather than the presence of an active infection. The levels of antibodies against PknG measured using a serum ELISA support this statement. However, further evaluation is necessary due to the bimodal immune response to MAP infection. The onset of MAP infection includes a cell-mediated response, which is characterized by the release of proinflammatory mediators, such as IFN-*γ* (T_H_1 response), and slowly increasing amounts of MAP-specific antibodies from B cells [33–35]. As the infection progresses high levels of antibodies are produced [36], but a shift to a chronic disease state results in progressive T cell exhaustion characterized by immunological dysfunction and improper control of the disease [36, 37]. Similar evidence exists in animals where a chronic MAP infection leads to anergy [36]. It is worth mentioning that MAP may also regulate regulatory T cell (T_reg_) populations [38], as a decrease of their activity has been recently reported in ileal lesions associated with JD [39].

In conclusion, PknG is an enzymatically active kinase that is secreted during macrophage infection. The combination of these findings suggests that PknG may similarly confer intracellular survival to the pathogen by preventing phagosome–lysosome fusion. From our work and others it is of interest to further investigate PknG as a drug target [32, 40] and biomarker for serological testing. Future research should focus on identifying host substrate(s) of PknG, which would likely identify the corresponding signal transduction pathways modulated by this interaction and clarify the role of PknG in mycobacterial physiology and pathogenesis.

## Supplementary Materials

Supplementary Materials Supplementary information about a description of materials and methods accompanying this study.
