# Supplementary material for: Protein Kinase G Induces an Immune Response in Cows Exposed to Mycobacterium avium Subsp. paratuberculosis
Source: Biomed Res Int. 2018 Jan 18;2018:1450828. doi: 10.1155/2018/1450828 (PMC5822771; doi:10.1155/2018/1450828)
Supplement: Supplementary Materials — Supplementary information about a description of materials and methods accompanying this study. [file 1450828.f1.docx]

**SUPPLEMENTARY INFORMATION**

**Protein Kinase G induces an immune response in cows exposed to *Mycobacterium avium* subsp. *partuberculosis***

Horacio Bach^1,*^, Melissa Richard-Greenblatt ^1^, Eviatar Bach^1^, Marcelo Chaffer^2^, Wanika Lai^1^, Greg Keefe,^2^ and Douglas J Begg^3^

^1^ Department of Medicine, Division of Infectious Diseases, University of British Columbia, 410-2660 Oak St., Vancouver, V6H 3Z6, BC, Canada

^2^ Department of Health Management, Atlantic Veterinary College, University of Prince Edward Island, 550 University Avenue, Charlottetown, C1A 4P3, PEI, Canada

^3^ Sydney School of Veterinary Science, School of Life and Environmental Sciences, The University of Sydney, 425 Werombi Rd, Camden, 2570, NSW, Australia

**1. Materials and methods**

*1.1 Production of recombinant PknG*

Recombinant PknG was produced in *E. coli* BL21 cells as follows: a starter culture was prepared by inoculating a single colony in LB supplemented with kanamycin and placed in a shaker at 37^o^C overnight. After 16 h, the starter was diluted 1:100 in fresh LB medium supplemented with kanamycin and cultured in the shaker at the same temperature. When the culture reached an optical density of 0.6, cells were induced by the addition of 0.4 mM IPTG (Invitrogen), and incubated at room temperature overnight. The following day, bacteria were harvested by centrifugation at 6,000 × *g* for 10 min, and resuspended in lysis buffer (50 mM NaH_2_PO_4_, 300 mM NaCl, 10 mM imidazole, pH 8.0) and stored at −20ºC until further use. Purification of PknG was carried out by affinity chromatography using Ni-NTA resin (Qiagen) in accordance to manufacturer instructions. Frozen cells in the lysis buffer were thawed in a cold-water bath then sonicated at 90 W for 20 seconds (three times). The lysate was centrifuged for 30 min at 27,000 × *g*. The supernatant was loaded onto the Ni-NTA resin column pre-equilibrated with the lysis buffer. The column was washed using washing buffer (50 mM NaH_2_PO_4_, 300 mM NaCl, 20 mM imidazole, pH 8.0) and PknG was eluted in elution buffer (50 mM NaH_2_PO_4_, 300 mM NaCl, 250 mM imidazole, pH 8.0). Immediately following elution, the protein was desalted in a PD-10 column (GE Healthcare) using 20 mM Tris-HCl, pH 7.4 supplemented with 20% glycerol.

*1.2. THP-1 cells infection*

THP-1 cells were washed with RPMI and seeded in 24-well plates (Corning Inc., Corning, NY) at a density of 2.5 x 10^5^ cells/well. THP-1 cells were differentiated into a macrophage-like cell line with 20 ng/ml phorbol myristate acetate. Strains were incubated at 37°C in a humidified atmosphere of 5% CO_2_ for 18 h.

Following differentiation, THP-1 cells were infected with exponentially growing MAP (OD_600_ = 0.5) at an MOI of 5:1. Macrophages were incubated with MAP for 3 h at 37ºC, 5% CO_2_. Wells were washed three times and resuspended in culture medium containing 100 μg/mL gentamicin to kill any remaining extracellular MAP*.* For colony forming unit (CFU) counting, cells were further washed with incomplete culture medium and the macrophages were lysed using 0.025% SDS at the selected time points post-infection. Serial dilutions of the lysate were plated onto Middlebrook 7H10 agar medium supplemented with OADC and mycobactin J. Colonies were counted when they were visible after an incubation time of at least 8 weeks at 37ºC.

*1.3. Immunostaining and fluorescence microscopy*

Macrophages (2.5 x 10^5^ cells/well) were differentiated on cover slips and fixed with 2.5% *p*-formaldehyde for 30 min at ambient temperature after the infection with opsonized MAP as detailed above. After washing with Hank’s buffer, cover slips were incubated for 15 min with sodium borohydrate (0.05% PBS wt/vol). Samples were blocked with 10% goat serum (prepared in Hank’s buffer) for 30 min and then incubated with 10% saponin in PBS containing 10% goat serum. After 20 min, polyclonal anti-PknG antibody (1:1,000) (raised against Mtb PknG and kindly provided by Dr. Yossef Av-Gay, University of British Columbia, Vancouver, Canada) was permeabilized for 30 min, followed by goat-anti-rabbit antibody conjugated to Alexa 488 (Invitrogen) (1:1,000) and examined by fluorescence microscopy using an epifluorescence microscope (Zeiss Axioplan II), and according to previously described protocol [1].

*2.5. THP-1 cell lysate immunoprecipitation*

At 72 h post-infection, 30 cell culture dishes containing MAP-infected macrophages were scraped from plates, harvested by centrifugation at 2,000 × *g* for 10 min, and lyzed by using an hypotonic solution (10 mM NaCl and 20 mM Tris-HCl, pH 7.5) for 30 min [2]. Cell debris was discarded after centrifugation. After filtration using a 0.22 μm-pore-size filter, the cell-free lysate was incubated with 100 μL polyclonal rabbit anti-PknG antibodies for 2 h at room temperature, and purified using a Protein G resin following the manufacturer’s instructions (Amersham) [1].

**REFERENCES**

1. H. Bach, K.G. Papavinasasundaram, D. Wong, Z. Hmama, and Y. Av-Gay, “*Mycobacterium* *tuberculosis* virulence is mediated by PtpA dephosphorylation of human vacuolar protein sorting 33B.” *Cell Host and Microbe*, vol. 3, no. 5, pp. 316–322, 2008.

2. H. Bach, J. Sun, Z. Hmama, and Y. Av-Gay, “*Mycobacterium avium* subsp. *paratuberculosis* PtpA is an endogenous tyrosine phosphatase secreted during infection.” *Infection and Immunity*, vol. 74, no. 12, pp. 6540–6546, 2006.
